# Bacterial‐Mediated Tumor Therapy: Old Treatment in a New Context

**DOI:** 10.1002/advs.202205641

**Published:** 2023-03-12

**Authors:** Yao Liu, Lili Niu, Nannan Li, Yang Wang, Mingyang Liu, Xiaomin Su, Xuhui Bao, Bo Yin, Shun Shen

**Affiliations:** ^1^ Key Laboratory of Spine and Spinal Cord Injury Repair and Regeneration of Ministry of Education Orthopaedic Department of Tongji Hospital, The Institute for Biomedical Engineering and Nano Science Tongji University School of Medicine Shanghai 200092 P. R. China; ^2^ Pharmacy Department and Center for Medical Research and Innovation Shanghai Pudong Hospital Fudan University Pudong Medical Center Shanghai 201399 China; ^3^ Central Laboratory First Affiliated Hospital Institute (College) of Integrative Medicine Dalian Medical University Dalian 116021 China; ^4^ Department of Surgical Oncology and General Surgery The First Hospital of China Medical University 155 North Nanjing Street, Heping District Shenyang 110001 China; ^5^ Institute for Therapeutic Cancer Vaccines Fudan University Pudong Medical Center Shanghai 201399 China; ^6^ Institute for Therapeutic Cancer Vaccines and Department of Oncology Fudan University Pudong Medical Center Shanghai 201399 China

**Keywords:** bacteria, bacterial components, engineered bacteria, immunotherapy, tumor microenvironment

## Abstract

Targeted therapy and immunotherapy have brought hopes for precision cancer treatment. However, complex physiological barriers and tumor immunosuppression result in poor efficacy, side effects, and resistance to antitumor therapies. Bacteria‐mediated antitumor therapy provides new options to address these challenges. Thanks to their special characteristics, bacteria have excellent ability to destroy tumor cells from the inside and induce innate and adaptive antitumor immune responses. Furthermore, bacterial components, including bacterial vesicles, spores, toxins, metabolites, and other active substances, similarly inherit their unique targeting properties and antitumor capabilities. Bacteria and their accessory products can even be reprogrammed to produce and deliver antitumor agents according to clinical needs. This review first discusses the role of different bacteria in the development of tumorigenesis and the latest advances in bacteria‐based delivery platforms and the existing obstacles for application. Moreover, the prospect and challenges of clinical transformation of engineered bacteria are also summarized.

## Graphical abstract

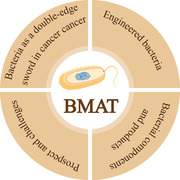

## 1 Introduction

Cancer is the second most common cause of death among children and adults.^[^ ^1^ ^]^ In 2020, an estimated 19.3 million new cancer cases (18.1 million excluding nonmelanoma skin cancer) and almost 10.0 million cancer deaths (9.9 million excluding nonmelanoma skin cancer) occurred in worldwide.^[^ ^2^ ^]^ The existence of the blood–brain barrier^[^ ^3^ ^]^ and the blood–tumor barrier^[^ ^4^ ^]^ greatly limits drug delivery to tumor tissue. Multiple biological barriers exist in tumor tissues, such as dense extracellular matrix and high interstitial pressure, resulting in low drug release capacity and therapeutic efficiency.^[^ ^5^ ^]^ Therefore, it is urgent to explore innovative approaches to improve antitumor efficacy and reduce adverse effect for cancer treatment.^[^ ^6^ ^]^

In 1813, Vautier discovered that patients infected with *Clostridium perfringens* (*C. perfringens*) had tumor regression.^[^ ^7^ ^]^ In 1893, Coley discovered that *Streptococcus pyogenes* could actually result in fibrosarcoma regression.^[^ ^8^ ^]^ Although bacteria‐mediated antitumor therapy (BMAT) appeared earlier than radiotherapy or chemotherapy,^[^ ^9^ ^]^ the inability to control bacterial infection limited its clinical application at that time. From 1990s, as researchers gained a more comprehensive understanding of the tumor microenvironment (TME) and tumor immunology as well as the development of numerous antibiotics, many studies on genetic engineering and synthetic biology were carried out to examine the application of live bacteria for cancer therapy.^[^ ^10^ ^]^ Attenuated bacteria with inherent tumor‐colonizing capacity have been rationally designed for tumor‐targeted delivery of a variety of different compounds to enhance bacteria‐based cancer combination therapy.^[^ ^11^ ^]^

**Figure 1:**
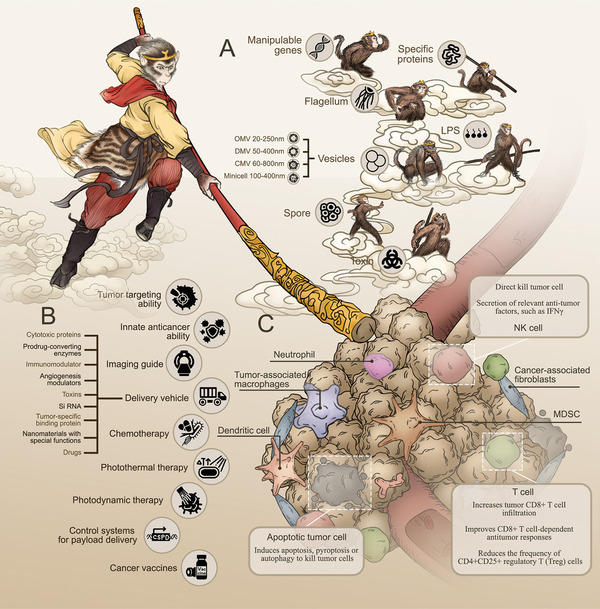
An overview of bacteria‐based cancer therapy. A) Bacteria have many natural advantages that can be applied to cancer therapy, such as manipulate genes, flagella that push forward, special proteins, toxins that can directly kill tumors. Bacterial spores and various derived vesicles can also be used for cancer therapy. B) Bacteria have tumor targeting, innate anticancer, and carrier function, and can be combined with chemotherapy, photodynamic therapy, photothermal therapy, immunotherapy, etc. C) Mechanisms of tumor cell death by tumor‐targeting bacteria: directly kill tumor cells or improve tumor immunosuppressive microenvironment.

Compared with traditional therapies, BMAT has many advantages (**Figure** **1**). 1) Tumor targeting:^[^ ^12^ ^]^ the hypoxic TME can preferentially induce bacterial colonization, while the necrotic tumor center provides abundant nutrients for bacterial growth and reproduction.^[^ ^9^, ^13^ ^]^ 2) Intratumoral penetration: the bacterial flagella allow them move autonomously to penetrate the tumor mass.^[^ ^14^ ^]^ 3) Tumor colonization, immune suppression in the tumor microenvironment allowed these bacteria to accumulate in tumors, but it will be eliminated by the immune system in normal tissues.^[^ ^15^ ^]^ 4) Bacterial cytotoxicity for tumor killing,^[^ ^16^ ^]^ which would allow the use of more toxic molecules without systemic effects.^[^ ^17^ ^]^ 5) Convenient genetic manipulation allows bacteria to be engineered for drug deliver with spatial and temporal precision, enabling locally higher concentrations of therapeutics whereas preventing potential toxicity due to systemic administration.^[^ ^18^ ^]^ 6) The choice of a commensal or probiotic bacterial strain will help ensure biocompatibility.^[^ ^19^ ^]^ 7) Specific proteins and surface markers can enable the visualization of the delivery of antitumor therapies by imaging the abundant proteins on the bacterial surface allow them to be combined with a variety of therapies.^[^ ^20^ ^]^ 8) Bacteria and various derivatives can regulate tumorigenesis and induce strong antitumor immunity.^[^ ^21^ ^]^ Herein, we aim to present a comprehensive overview of BMAT. First, we focus on the relationship and role of bacteria in carcinogenesis; second, we summarize the application and development of BMAT; last but not least, we discuss the challenges and future directions for BMAT.

## 2 Bacteria as a Double‐Edge Sword in Cancer

In 2020, a study reported that certain types of tumor have their own unique microbial composition,^[^ ^22^ ^]^ which has an important role in the survival of cancer patients.^[^ ^23^ ^]^ Undeniably, host microbiota is a crucial mediator in modulating cancer development and progression in addition to the well‐known genetic, epigenetic, and stromal microenvironment regulators.^[^ ^24^ ^]^ Therefore, a comprehensive understanding of bacteria in the tumor evolution can provide novel strategies for the prevention, diagnosis, and treatment for cancers. This section will comprehensively describe the role of bacteria in the carcinogenesis and development of tumors from three aspects: symbiotic bacteria, pathogenic bacteria, and treatable bacteria.

### 2.1 The Relationship between Commensal Bacteria and Tumors

The commensal microbiota represents the microorganisms that live in close contact with the host, which is fundamental for the host fitness.^[^ ^25^ ^]^ Trillions of microbes inhabit the mouth, lung, gut, skin, etc.^[^ ^26^ ^]^ When dysbiosis occurs, the number of pathogenic bacteria increases,^[^ ^27^ ^]^ leading to the occurrence and development of many diseases including cancer. For example, the lung microbiota dysregulation will stimulate tissue‐resident *γδ* T cells produced IL‐17 and other proinflammatory mediators to promote neutrophil expansion and tumor cell proliferation.^[^ ^28^ ^]^ Gut initial structure and microbiota cometabolism of the microbiome determines susceptibility to tumorigenesis,^[^ ^29^ ^]^ which may also indirectly affect the occurrence of lung cancer through systemic immune responses.^[^ ^28^ ^]^ These commensal microorganisms and metabolites can also mediate immune activation in cancer chemotherapy.^[^ ^30^ ^]^ Microbial diversity has been shown to be correlated with immunological signatures of the TME,^[^ ^23^ ^]^ while fecal bacterial‐specific markers can also be used to diagnose and prognosis tumors.^[^ ^31^ ^]^ These have been verified in prostate cancer^[^ ^25^ ^]^ and pancreatic ductal adenocarcinoma (PDAC) patients. Further, gut microbiota can form barrier protection by competing for epithelial adhesion sites and nutrients, as well as participating in pathogen competition and tissue repair.^[^ ^32^ ^]^ Therefore, delivery of probiotics to the host microbiota is a promising method to prevent and treat many diseases.^[^ ^33^ ^]^

Although promoting the proliferation and development of beneficial bacteria and inhibiting the growth and reproduction of harmful bacteria by regulating the homeostasis of symbiotic bacteria seems to be a promising method to treat cancers,^[^ ^28^ ^]^ the study of symbiotic microorganisms also has some obstacles to overcome. First, a major obstacle to profile gut microbiome as a cancer biomarker is stool sample collection.^[^ ^26c^ ^]^ Second, it is currently unclear whether assessment of the human microbiome from other types of biospecimens may also serve as important cancer biomarkers. Third, low biomass microbiome research requires strict experimental control to prevent contamination. At last, the source, type, and abundance of specific bacterial DNA in blood have not yet well characterized. In the future, more studies should be warranted to extensively investigate all the above aspects to initiate the further clinical use of those commensal bacteria for cancer diagnosis and treatment.

### 2.2 The Pro‐Oncogenic Role of Specific Bacteria in Tumorigenesis

Tumor is a new organism formed by abnormal clonal proliferation of some certain cells in the local tissue under the action of various carcinogenic factors.^[^ ^34^ ^]^ Its occurrence is a multifactor and multistep complicated process, which is closely related to infection,^[^ ^35^ ^]^ occupational exposure,^[^ ^36^ ^]^ environmental pollution, unreasonable diet,^[^ ^37^ ^]^ and genetic factors.^[^ ^38^ ^]^ Among them, ≈18% of cancers are attributable to infections.^[^ ^39^ ^]^ So far, many bacteria have been demonstrated to be tightly associated with various malignancies: *Salmonella typhimurium* (*S. typhimurium*) with hepatobiliary carcinoma,^[^ ^40^ ^]^ *Citrobacter rodentium*,^[^ ^41^ ^]^ *Streptococcus gallolyticus*,^[^ ^26a^ ^]^ *Peptostreptococcus anaerobius* (*P. anaerobius*),^[^ ^42^ ^]^ and *Clostridium difficile* (*C. difficile*)^[^ ^43^ ^]^ with colorectal cancer (CRC), *Mycobacterium tuberculosis* with lung cancer,^[^ ^44^ ^]^ *Fusobacterium nucleatum* (*F. nucleatum*) with CRC and pancreatic cancer,^[^ ^35^, ^45^ ^]^ *Porphyromonas gingivalis* (*P. gingivalis*) with oral squamous cell carcinoma.^[^ ^46^ ^]^ In general, bacteria may lead to malignant tumors by altering normal physiological processes of the host body through the following ways: 1) stimulating tumor cell proliferation and regulating the host‐cell viability;^[^ ^24^ ^]^ 2) increasing reactive oxygen species (ROS) and immune dysregulation caused by chronic inflammation;^[^ ^47^ ^]^ 3) downregulating tumor‐suppressive genes while upregulating oncogenic genes;^[^ ^39^ ^]^ 4) recruiting tumor‐infiltrating suppressive cells to antagonize antitumor immunity;^[^ ^48^ ^]^ 5) activating inflammasomes to generates a favorable microenvironment for tumor progression;^[^ ^48^ ^]^ and 6) affecting the efficacy of chemotherapy and immunotherapy.^[^ ^49^ ^]^ Their common pathogenic types and mechanism are shown in **Table** **1**.

**Table 1:** Examples of carcinogenic bacteria

Species	Cancer	Pathogenic mechanism
*F. nucleatum*	Breast cancer,^[^ ^50^ ^]^ CRC,^[^ ^30^, ^35^, ^51^ ^]^ cervical cancer,^[^ ^52^ ^]^ oral squamous cell carcinoma^[^ ^46^, ^53^ ^]^	Downregulating antitumor T‐cell mediated adaptive immunity;^[^ ^51d^ ^]^ initiating overexpressed NLRP3 (NOD‐like receptor thermal protein domain associated protein 3) and activating upstream signal molecules of ATR‐CHK1;^[^ ^53b^ ^]^ Fap2 protein of *F. nucleatum* directly interacted with TIGIT, leading to the inhibition of NK cell cytotoxicity;^[^ ^51e^ ^]^ miR‐1322/CCL20 axis and M2 polarization;^[^ ^35b^ ^]^ ANGPTL4‐mediated glycolysis;^[^ ^30^ ^]^ modulating E‐cadherin/*β*‐catenin signaling via its FadA adhesin.^[^ ^51c^ ^]^
*Helicobacter pylori*	Gastric carcinoma^[^ ^54^ ^]^	Preventing UsF1/p53 nuclear built up and relocating these complexes in the cytoplasm;^[^ ^54b^ ^]^ inhibiting miR‐373 synthesis;^[^ ^55^ ^]^ inhibiting p53 stress response via activation of Erk1/2‐HDM2‐p53 pathway;^[^ ^56^ ^]^ damaging DNA via NF‐*κ*B (nuclear factor‐kB).^[^ ^57^ ^]^
*Hungatella hathewayi*	CRC^[^ ^58^ ^]^	Upregulating DNA methyltransferase.^[^ ^58^ ^]^
*Eubacterium*	CRC^[^ ^45^ ^]^	Activating the transcription factor NF‐*κ*Β by endotoxin.^[^ ^45^ ^]^
*Chlamydia trachomatis*	Ovarian cancer,^[^ ^59^ ^]^ cervical cancer,^[^ ^60^ ^]^ squamous cell carcinoma^[^ ^61^ ^]^	Serotype G,^[^ ^61a^ ^]^ MMP‐9/RECK imbalance.^[^ ^61b^ ^]^
*Neisseria gonorrhoeae*	Ovarian cancer,^[^ ^59d^ ^]^ bladder cancer^[^ ^62^ ^]^	G1 arrest in human epithelial cells.^[^ ^63^ ^]^
*P. gingivalis*	Oral squamous cell carcinoma,^[^ ^46^, ^53^ ^]^ pancreatic^[^ ^64^ ^]^	Initiating overexpressed NLRP3 and activating upstream signal molecules of ATR‐CHK1,^[^ ^53b^ ^]^ interacting with oral epithelial cells through toll‐like receptors.^[^ ^46^ ^]^
*S. typhimurium*	Colon cancer,^[^ ^65^ ^]^ hepatobiliary carcinoma^[^ ^40^ ^]^	Activating *β*‐catenin^[^ ^66^ ^]^ and STAT3 signaling pathway^[^ ^67^ ^]^ and enhancing tumorigenesis.
*Mycobacterium tuberculosis*	Lung cancer^[^ ^68^ ^]^	Releasing the NO;^[^ ^69^ ^]^ upregulating P53 and Bcl‐2.^[^ ^70^ ^]^
*Campylobacter jejuni*	CRC,^[^ ^71^ ^]^ small intestinal lymphomas^[^ ^72^ ^]^	Promoting CRC through the genotoxic action of cytolethal distending toxin.^[^ ^71^ ^]^
*Propionibacterium acnes*	Prostate cancer^[^ ^73^ ^]^	Promoting M2 polarization of macrophages via TLR4/PI3K/Akt signaling.^[^ ^73a^ ^]^
*Chlamydia psittaci*	Ocular lymphomas^[^ ^74^ ^]^	NA
*Bacteroides fragilis*	CRC^[^ ^47^, ^75^ ^]^	Promoting proliferation of CRC via CCL3‐related molecular pathways,^[^ ^75c^ ^]^ the RHEB/mTOR pathway,^[^ ^76^ ^]^ mucosal *bft* exposure,^[^ ^77^ ^]^ and spermine oxidase overexpresse.^[^ ^47^ ^]^
*Streptococcus gallolyticus*	CRC^[^ ^26^, ^75^, ^78^ ^]^	Inducing mRNA expression of proinflammatory cytokines, IL‐1 and COX‐2, as well as angiogenic chemokine, IL‐8.^[^ ^78^ ^]^
*Helicobacter hepaticus*	Hepatocellular carcinoma (HCC),^[^ ^79^ ^]^ mammary tumors^[^ ^80^ ^]^	Activating Wnt/*β*‐catenin pathway,^[^ ^80^ ^]^ activating NF‐kB‐regulated networks associated with innate and T helper 1 (Th1)‐type adaptive immunity.^[^ ^81^ ^]^
*Veillonella*	Lung cancer^[^ ^82^ ^]^	Activating checkpoint inhibitor markers,^[^ ^82a^ ^]^ upregulating PI3K pathway.^[^ ^82c^ ^]^
*E. coli*	CRC,^[^ ^41^, ^83^ ^]^ bladder cancer^[^ ^84^ ^]^	Fostering bladder cancer cell line progression via epithelial mesenchymal transition, stemness, and metabolic reprogramming.^[^ ^84^ ^]^
*P. anaerobius*	CRC^[^ ^42^ ^]^	Interacting with integrin *α*_2_/*β*_1_ overexpressed on CRC cells.^[^ ^42b^ ^]^
*C. difficile*	CRC^[^ ^43^ ^]^	Depending on the *C. difficile* toxin TcdB.^[^ ^43^ ^]^

### 2.3 The Anti‐Oncogenic Role of Specific Bacteria in Tumorigenesis

TME refers to the surrounding microenvironment in which tumor cells reside with surrounding blood vessels,^[^ ^85^ ^]^ immune cells,^[^ ^86^ ^]^ fibroblasts,^[^ ^87^ ^]^ bone marrow‐derived inflammatory cells,^[^ ^88^ ^]^ various signaling molecules,^[^ ^89^ ^]^ and extracellular matrix.^[^ ^84^ ^]^ The rapid proliferation of tumor cells leads to the immature vascular structure inside the tumor center, which makes a hypoxic TME.^[^ ^90^ ^]^ Meanwhile, dense fibroblasts and high interstitial pressure further hinder the tissue penetration of many antitumor drugs,^[^ ^87a^ ^]^ while some specific anaerobes are able to invade and colonize hypoxic TME.^[^ ^91^ ^]^ Although many locally infected bacteria may have some common antitumor activities, different strains, tumor types, and even stages of bacteria‐host interactions may employ other unique mechanisms to eliminate cancer. Some bacteria can not only directly kill tumor cells, but also induce innate and adaptive immune responses against tumor‐infected bacteria and tumor cells, such as *S. Typhimurium*,^[^ ^9^, ^11^, ^92^ ^]^ *Listeria. monocytogenes* (*L. monocytogenes*), *Clostridium. novyi‐NT* (*C. novyi‐NT*, removal of the *α*‐toxin gene) spores,^[^ ^15^, ^93^ ^]^ etc. Bacteria with low cytotoxic to tumor cells can also regulate the systemic antitumor immunity, such as *Bifidobacterium infantis* (*B. infantis*).^[^ ^10^, ^94^ ^]^ With many comprehensive studies of immunology and synthetic biology, bacterial therapy has been widely investigated at the preclinical and clinical level. **Table** **2** summarizes former and recent clinical trials of BMAT. Besides, genetic engineering and/or biochemical synthesis can further reduce bacterial toxicity while enhance antitumor activities, making them a potential multifunctional platform that can be personalized to meet clinical therapeutic requirements. **Table** **3** summarizes examples of bacterial therapies in animal models.

**Table 2:** Clinical trials of BMAT

Species	Phase	Cancer type		Result	Refs.
*S. Typhimurium* VNP 20009	I	Metastatic melanoma (*n* = 24), metastatic renal cell carcinoma (*n* = 1)	Intravenously	Safely administered, and some tumor colonization	[95]
VNP 20009	I	Advanced unspecified solid tumors	Intravenously	N/A	NCT00006254^a)^
VNP 20009	I	Advanced or metastatic cancer (*n* = 45)	Intravenously	N/A	NCT00004988^a)^
*S. typhimurium* expressing cytosine deaminase (TAPET‐CD, VNP20029)	I	Three patients with advanced and metastatic solid tumors	Intratumoral injection	Tumor colonization	[96]
*C. novyi‐*NT spores	I	Retroperitoneal leiomyosarcoma (*n* = 1)	Intratumoral injection	Reduced the tumor within and surrounding the bone	[97] NCT01924689^a)^
*C. novyi‐*NT spores	I	Malignant neoplasm (*n* = 18)	Interventional	Recruiting	NCT03435952^a)^
*C. novyi‐*NT spores	I	Solid tumor malignancies (*n* = 5)	Interventional	Terminated	NCT01118819^a)^
*L. monocytogenes* (ANZ‐100 and CRS‐207)	–	Solid tumors (liver, pancreas, lung, or ovary) (*n* = 26)	Intravenously	Administration was safe and resulted in immune activation	[98]
*L. monocytogenes (CRS‐207)*	II	Pancreatic cancer (*n* = 90)	Intravenously	Extended survival for patients with minimal toxicity	[99]
*Bacillus‐Calmette ‐Guerin (BCG)*	*–*	Bladder cancer	Intravesical	Reduces the risk of shortand long‐term relapse	[100]
*BCG*	I	Transitional cell carcinoma (*n* = 6)	Intradermally	Well tolerated, T‐cell responses	[101] NCT00070070^a)^
*BCG*	II	Bladder cancer (*n* = 32)	Intravesical	Reducing the recurrence and prolonging survival	NCT02015104^a)^

^a)^ ClinicalTrials.gov.

**Table 3:** Examples of bacterial therapies in animal models

Species	Experimental model	Route of administration	Result
*L. monocytogenes*	CT26 colon tumor lung metastases,^[^ ^102^ ^]^ CT26 colon tumor,^[^ ^93a^ ^]^ pancreatic cancer,^[^ ^15^, ^93^, ^99^, ^103^ ^]^ metastatic breast cancer,^[^ ^15^, ^104^ ^]^ TC‐1 mouse tumor,^[^ ^105^ ^]^ HCC,^[^ ^106^ ^]^ PDAC^[^ ^107^ ^]^	i.v. i.p.	Induced potent and durable effector and memory T‐cell responses;^[^ ^102^ ^]^ eradicated the metastases and suppressed the growth of pancreatic cancer;^[^ ^15^, ^103^, ^107^ ^]^ significantly reduced regulatory T cells (Treg) and myeloid‐derived suppressor cells (MDSC);^[^ ^93^, ^104^, ^105^ ^]^ extended survival of pancreatic patients,^[^ ^99^ ^]^ enhanced specific CD81 T‐cell activity.^[^ ^106^ ^]^
*Enterococcus hirae*	MCA205 sarcoma^[^ ^108^ ^]^	i.g.	Activated the autophagy machinery in enterocytes.^[^ ^108^ ^]^
*S. typhimurium*	Metastatic melanoma,^[^ ^95^ ^]^ melanoma,^[^ ^109^ ^]^ PC‐3 prostate cancer,^[^ ^12^, ^110^ ^]^ breast tumor,^[^ ^109^, ^111^ ^]^ HCC,^[^ ^112^ ^]^ colon carcinomas,^[^ ^109^, ^111^, ^112^, ^113^ ^]^ CT26 colon cancer,^[^ ^114^ ^]^ MC38 colon cancer,^[^ ^115^ ^]^ schwannoma,^[^ ^116^ ^]^ bladder cancer,^[^ ^92^, ^117^ ^]^ PDAC,^[^ ^12^, ^118^ ^]^ fibrosarcoma^[^ ^119^ ^]^	i.v. i.t.	Tumor regression;^[^ ^95^, ^109^ ^]^ selectively killed tumor cells with high efficacy;^[^ ^12a^ ^]^ expressing fas ligand;^[^ ^111b^ ^]^ selectively targeted metastases;^[^ ^111d^ ^]^ decreased tumor angiogenesis;^[^ ^116^ ^]^ decreased the lung metastases;^[^ ^112b^ ^]^ suppressed tumor growth via the proinflammatory cytokine interleukin‐1*β*;^[^ ^114a^ ^]^ activated intratumoral M1 macrophages and reduced M2 macrophages;^[^ ^115^ ^]^ secreted ClyA into the TME.^[^ ^12c^ ^]^
*E. coli*	4T1 breast tumors,^[^ ^120^ ^]^ CT26 colon cancer,^[^ ^20^, ^121^ ^]^ MC38 colon cancer,^[^ ^122^ ^]^ MCF7 breast tumors,^[^ ^123^ ^]^ human LS174T colon adenocarcinoma,^[^ ^124^ ^]^ A20 lymphoma^[^ ^125^ ^]^	i.v. p.o.	Tumor targeting and macrophage polarization;^[^ ^120c^ ^]^ reduced pulmonary metastases;^[^ ^120a^ ^]^ tumor regression and necrosis;^[^ ^123^ ^]^ produced high local concentrations of arginine.^[^ ^122^ ^]^
*B. infantis*	Lewis lung cancer (LLC),^[^ ^94a^ ^]^ melanoma,^[^ ^32^ ^]^ MC38 colon cancer^[^ ^126^ ^]^	i.v. p.o. i.t.	Inhibited tumor growth and prolonged survival time of LLC C57BL/6 mice;^[^ ^94a^ ^]^ facilitated CD47‐based immunotherapy via STING signaling.^[^ ^126^ ^]^
*Bifidobacterium bifidum (B. bifidum)*	MC38 colon cancer^[^ ^94b^ ^]^	i.g.	Enhanced biosynthesis of immune‐stimulating molecules and metabolites.^[^ ^94b^ ^]^
*Streptococcus thermophilus*	CRC^[^ ^127^ ^]^	i.g.	Inhibited colorectal tumorigenesis through secreting *β*‐galactosidase.^[^ ^127^ ^]^
*C. novyi‐*NT spores	HCT116 colon cancer,^[^ ^128^ ^]^ B16 melanoma,^[^ ^128^ ^]^ CRC^[^ ^129^ ^]^	i.v.	Rapid and dramatic regressions of experimental tumors in mice;^[^ ^128^ ^]^ inhibited the development of DMH‐induced CRC.^[^ ^129^ ^]^
*Fusobacterium nucleatum*	CRC^[^ ^130^ ^]^	i.g.	Improved therapeutic responses to PD‐1 (programmed cell death protein‐1) blockade.^[^ ^130^ ^]^
*Lactobacillus gallinarum*	CRC^[^ ^131^ ^]^	i.g.	Produced protective metabolites that can promote apoptosis of CRC cells.^[^ ^131^ ^]^
*Lactobacillus coryniformis*	Colitis‐associated (CA)‐CRC mouse model^[^ ^132^ ^]^	i.g.	Ameliorated CA‐CRC via regulating intestinal microenvironment, alleviated inflammation, and intestinal barrier damage.^[^ ^132^ ^]^
*Lactobacillus plantarum*	Oral cancer^[^ ^133^ ^]^	N/A	Induced apoptosis in oral cancer KB cells through upregulation of PTEN and downregulation of MAPK signaling pathways.^[^ ^133^ ^]^
*Lactobacillus reuteri*	Melanoma,^[^ ^134^ ^]^ CRC^[^ ^135^ ^]^	i.g.	Reduced cancer incidence and prolonged the survival of tumor‐bearing mice.^[^ ^134^ ^]^
*Lactobacillus. acidophilus*	4T1 breast cancer^[^ ^136^ ^]^	i.p.	Reduced tumor growth rate and increased lymphocyte proliferation.^[^ ^136^ ^]^
*Lactobacillus* *rhamnosus*	CT26 colon cancer,^[^ ^137^ ^]^ bladder tumor,^[^ ^138^ ^]^ DMH‐induced CRC,^[^ ^139^ ^]^ CA‐CRC^[^ ^137^, ^140^ ^]^	i.g.	Restored antibiotic‐disrupted gut microbiota;^[^ ^137^ ^]^ reduced tumor incidence, multiplicity and volume;^[^ ^139^ ^]^ ameliorated impaired microbiota.^[^ ^140a^ ^]^

## 3 Engineered Bacteria

Some bacteria are at risk of uncontrolled toxicity and proliferation.^[^ ^93a^ ^]^ Therefore, engineering bacterial is necessary and should focus on reducing bacterial toxicity,^[^ ^106^ ^]^ improving biocompatibility,^[^ ^141^ ^]^ and diversifying functional transformation.^[^ ^142^ ^]^ In this subsection, we summarize several approaches to bacterial engineering and their potential benefits and challenges for cancer treatment.

### 3.1 Construction of Bacteria/Nanomaterial Composites

Traditional nanomaterials have limited efficacy due to poor targeting ability, rapid recognition, and systemic toxicity. BMAT may be able to overcome these limitations. 1) Bacteria, as therapeutic agents, are modified by nanosynthesis technology to escape the immune phagocytic system.^[^ ^141^ ^]^ 2) Bacteria are used as carriers to realize multifunctional diagnosis and treatment for tumor by combining with other functional nanomaterials.^[^ ^143^ ^]^ 3) Integration of bacteria and functional nanomaterials to support each other.^[^ ^144^ ^]^ In a nutshell, whether bacteria serve as therapeutic agents, targeting carriers, and/or immune activators, which can significantly improve the antitumor effect and provide a robust potential to develop novel antitumor therapeutic strategies.

#### 3.1.1 Wear an Invisible Cloak for Bacteria

Although BMAT is attractive, bacteria are inevitably easily phagocytosed and cleared by the reticuloendothelial system after entering the host body.^[^ ^93a^ ^]^ Therefore, engineering bacteria through surface modification aims to extend their retention in circulation. Synthetic polymers,^[^ ^145^ ^]^ liposomes,^[^ ^146^ ^]^ and biofilms^[^ ^147^ ^]^ have been widely used in nanomedicine for surface modification to improve biocompatibility, targeting ability, and blood circulation. These materials can also be applied to the surface modification of bacteria.

**Figure 2:**
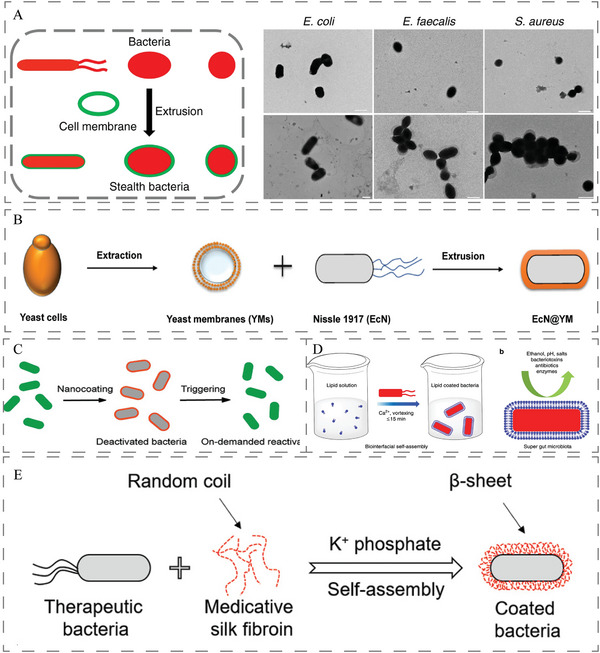
A) Schematic illustration for the preparation of CMCB by extruding bacteria with cell membranes. Reproduced with permission.^[^ ^141^ ^]^ Copyright 2019, Springer Nature. B) Preparation of EcN@YM by extruding probiotic EcN with extracted YMs through a polycarbonate porous membrane with an average pore size of 1 µm. Reproduced with permission.^[^ ^149^ ^]^ Copyright 2021, The American Association for the Advancement of Science. C) Bacterial deactivation by restraining inside a smart nanocoating and the on‐demand reactivation enabled by a biologically relevant stimulus‐triggered decoating. Reproduced with permission.^[^ ^157^ ^]^ Copyright 2020, Wiley‐VCH. D) Schematic illustration of the preparation of lipid membrane coated bacteria by biointerfacial supramolecular self‐assembly. Reproduced with permission.^[^ ^155^ ^]^ Copyright 2019, Springer Nature. E) Coating therapeutic bacteria with medicative silk fibroin by biointerfacial self‐assembly. Reproduced with permission.^[^ ^156^ ^]^ Copyright 2021, Wiley‐VCH.

As shown in **Figure** **2**A, Cao et al. coated erythrocyte membrane to *E. coli Nissle 1917* (EcN) (CMCB) by extrusion method.^[^ ^141^ ^]^ The strategy of generating stealth bacteria through cell membrane camouflage resulted in prolonged blood retention, reduced inflammatory response, and increased tumor accumulation after intravenous injection. Similarly, this camouflage strategy has also been applied to tumor delivery of *L. monocytogenes* ^[^ ^93a^ ^]^ and *P. gingivalis*.^[^ ^148^ ^]^ In addition, EcN camouflaged with yeast membranes (YMs) (EcN@YM) by physical coextrusion of porous membranes (Figure 2B) can be delivered to lymphoid follicles and promoted robust mucosal immune response.^[^ ^149^ ^]^ Membranes from various cells can also be applied, including red blood cells, platelets,^[^ ^150^ ^]^ macrophages,^[^ ^151^ ^]^ neutrophils,^[^ ^152^ ^]^ and cancer cells.^[^ ^153^ ^]^ Similarly, other bacterial strains can also apply this strategy, which will become a reliable method with higher safety and enhanced therapeutic efficacy.

Considering the fragility of cells, species specificity, centrifugation, temperature fluctuations, and other factors, the extraction process often cause giant variance of the cell membranes quality.^[^ ^154^ ^]^ Therefore, the surface of bacteria can be modified by chemical conjugation or physical encapsulation. For example, by simply codepositing with dopamine under biocompatible conditions, various functional small molecules and polymers can be simultaneously anchored to form multifunctional coatings on EcN surfaces (Figure 2C),^[^ ^154^ ^]^ which has also been verified on VPN20009.^[^ ^9a^ ^]^ Similarly, lipid membrane‐encapsulated bacteria could be rapidly prepared in less than 15 min by simply vortexing EcN with a biocompatible lipid (Figure 2D).^[^ ^155^ ^]^ To avoid using organic solvents and chemical reagents, silk fibroin can self‐assemble onto the bacterial surface through a transition from random coil to *β*‐sheet conformation. This layer‐by‐layer procedure seems to be easier to scramble (Figure 2E).^[^ ^156^ ^]^

Both biologically derived membrane coating and surface modification through chemical biosynthesis can enhance the ability of therapeutic bacteria to resist in vivo physicochemical factors and improve their therapeutic efficiency. At the same time, it may also confer additional functions on therapeutic bacteria, which we will introduce in the next section.

#### 3.1.2 Combined Treatment with Chemotherapy

The anticancer activities of chemotherapies rely on disrupting DNA integrity, enzymes for DNA repair and synthesis. Chemotherapy has limited efficacy due to therapeutic resistance and systemic toxicity, which prevents the use of more aggressive dosage.^[^ ^158^ ^]^ Bacteria‐based delivery systems may be able to overcome these limitations by autonomous propulsion and selective colonization of hypoxic tumor tissue in the host.^[^ ^111^, ^159^ ^]^ Therefore, in order to achieve more effective antitumor action, chemotherapy can be combined with bacteria‐based delivery systems.^[^ ^160^ ^]^

**Figure 3:**
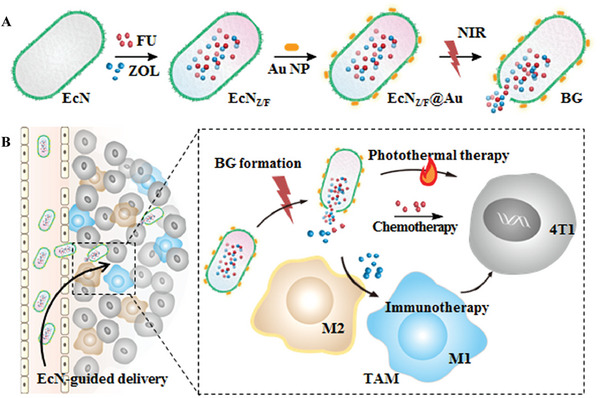
Preparation and therapeutic mechanism of EcN_Z/F_@Au/NIR. A) EcN_Z/F_@Au are prepared by inoculation of FU and ZOL into EcN through electroporation and decoration of Au NRs on the EcN surface. B) Self‐guided motion of EcN promotes extravasation of EcN_Z/F_@Au across blood vessels, accumulation in tumor tissues, and interaction with tumor cells. NIR illumination exhibits the photothermal effect on tumor cells and initiates the transformation into bacterial ghost. The local release of FU from bacterial ghost produces chemotherapeutic effect to tumor cells, while that of ZOL enhances the polarization of TAMs toward the M1 phenotype for immunotherapy. A,B) Reproduced with permission.^[^ ^163^ ^]^ Copyright 2021, Elsevier.

For example, Zoaby et al. loaded doxorubicin (DOX)‐containing liposomes into motile *S. typhimurium* VNP20009 via electroporation. The bacteria sense and invade the cancer cells, where the drug is released internally.^[^ ^159^ ^]^ Yang et al. developed an engineered bacteria delivery system (GDOX@HSEc) through attached DOX‐loaded glycogen nanoparticles (GDOX NPs) to the surface of engineering bacteria expressing heparin sulfatase 1(HSulf‐1),^[^ ^160^ ^]^ which significantly suppressed melanoma, with reduced side effects in murine models. Similarly, Xiao et al. employed anaerobic *B. infantis* to deliver adriamycin‐loaded bovine serum albumin nanoparticles (DOX‐NPs) (Bif@DOX‐NPs), significantly prolonging the median survival of the tumor‐bearing mice to 69 days and reducing the toxic side‐effects of DOX.^[^ ^161^ ^]^ Gwisai et al. integrated magnetic nanoparticles and nanoliposomes loaded with photothermal agents (indocyanine green (ICG)) and chemotherapeutic molecules (DOX) onto *E. coli* with an efficiency of ≈90% to form a magnetic controlled bacterial biological hybrid.^[^ ^162^ ^]^ Chemotherapeutic agent 5‐fluorouracil (FU) and macrophage phenotype regulator zoledronic acid (ZOL) were loaded into EcN through electroporation, followed by decoration of Au nanorods on the ECN surface to construct EcN_Z/F_@Au. An intermittent near‐infrared (NIR) illumination causes stepwise increases in the bacterial ghosts (BGs) formation and drug release. In addition to the self‐guided motion of EcN and chemotherapeutic effect of FU, the local release of ZOL enhances valid polarization of tumor‐associated macrophage (TAMs) toward the M1 phenotype and an effective production of proinflammatory cytokines, leading to a synergistic efficacy on 4T1 tumor growth inhibition (**Figure** **3**).^[^ ^163^ ^]^

#### 3.1.3 Combined Treatment with Virus

**Figure 4:**
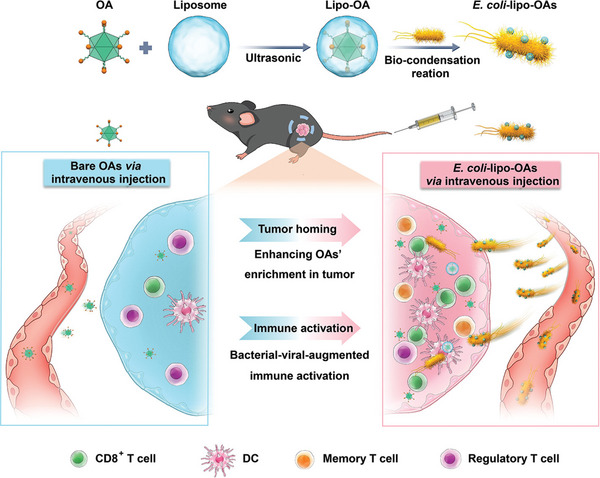
Schematic representation of self‐propelled bacterium‐vessels carrying OAs that can home to the tumor lesion and enhance the antitumor responses through bacterial–viral‐augmented immune activation. Reproduced with permission.^[^ ^165^ ^]^ Copyright 2022, American Chemical Society.

Oncolytic viruses (OVs) are considered non‐self and thus lead to the initiation of immune responses due to their natural adjuvant properties.^[^ ^164^ ^]^ To avoid low enrichment and limited immune activation, bacteria were also used for intratumoral delivery of OVs. As shown in **Figure** **4**, Sun et al. prepared liposomal oncolytic adenoviruses (OAs) (named lipo‐OAs) containing active ester use active ester‐bearing, two‐tailed lipids (1,2‐distearoyl‐sn‐glycero‐3‐phosphoethanolamine‐*N*‐methoxy‐ (polyethylene glycol)2000‐*N*‐hydroxysuccinimide, SPEPEG2000‐NHS) and protein lecithin. Followed by, the bonds between amines on the surfaces of *E. coli* BL21 and ester‐containing liposomal OAs are connected to form called *E. coli*‐lipo‐OAs.^[^ ^165^ ^]^ Notably, the enrichment of OAs transported by self‐propelled bacterial microbe vehicles from *E. coli*‐lipo‐OAs can be increased by more than 170‐fold compared to that of intravenously injected bare OAs in a nonsmall cell lung cancer model, which significantly enhanced antitumor immunity through bacterial–viral‐augmented immune responses.^[^ ^165^ ^]^

#### 3.1.4 Combined with Radiotherapy

**Figure 5:**
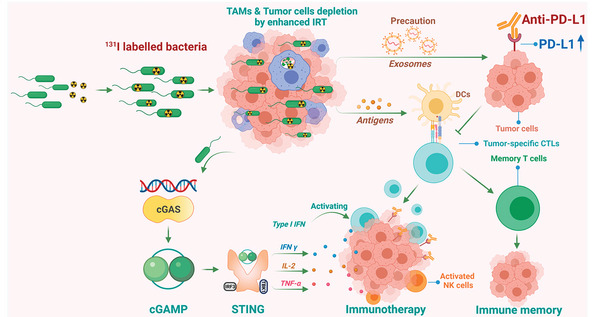
Mechanism of inactivated bacteria to boost multiple antitumor immune responses for radio‐immunotherapy. Reproduced with permission.^[^ ^166^ ^]^ Copyright 2022, American Chemical Society.

Live attenuated bacteria to selectively deliver radionuclides to metastases and tumors have been successfully used in many studies.^[^ ^15^, ^103^ ^]^ For example, both radioisotopes ^188^Rhenium^[^ ^103^ ^]^ and ^32^P^[^ ^15c^ ^]^ were coupled to an attenuated live *L. monocytogenes* to create a unique radioactive *Listeria* ^at^, which resulted in significant suppression of Panc‐02 tumor growth. As shown in **Figure** **5**, to perform radiolabeling, iodogen was first dissolved in dichloromethane and dried by nitrogen purging. Afterward, inactivated VNP20009 suspension in PBS was sufficiently mixed with 5 mCi of Na^131^I. The obtained mixture was then added into an EP tube containing iodogen for sufficient shaking at 37 °C. ^131^I‐labeled inactivated bacteria (^131^I‐VNP) were acquired after purification by centrifuging and washing with PBS three times. ^131^I‐VNP‐mediated local internal radioisotope therapy can further stimulate a robust systemic antitumor immune response by activating the cGAS‐STING pathway of innate immunity and promoting DC cell maturation for T‐cell‐dominated adaptive immunity. Combined with systemic checkpoint blockade therapy (antiprogrammed cell death–ligand 1, anti‐PD‐L1), ^131^I‐VNP combination therapy can further inhibit the growth of colon cancer in situ and prevent tumor recurrence and metastasis.^[^ ^166^ ^]^

#### 3.1.5 Combined with Photothermal Therapy

Photothermal therapy (PTT) is a method to ablate tumor by converting light energy into heat energy using photothermal agent stimulated by NIR.^[^ ^167^ ^]^ Unfortunately, the poor accumulation of photothermal agents at tumor sites limits therapeutic efficacy.^[^ ^168^ ^]^ In terms of the spatial specificity and non‐invasiveness of NIR, bacterial‐mediated drug delivery could well address this obstacle.^[^ ^9^, ^169^ ^]^ Currently, there are two main modes of bacteria‐based photothermal. One is that bacteria act as tumor‐specific photothermal agents without any chemical modification or loading of additional payloads. The other is that bacteria as carrier to transport photothermal agents to tumor sites through chemical synthesis.

**Figure 6:**
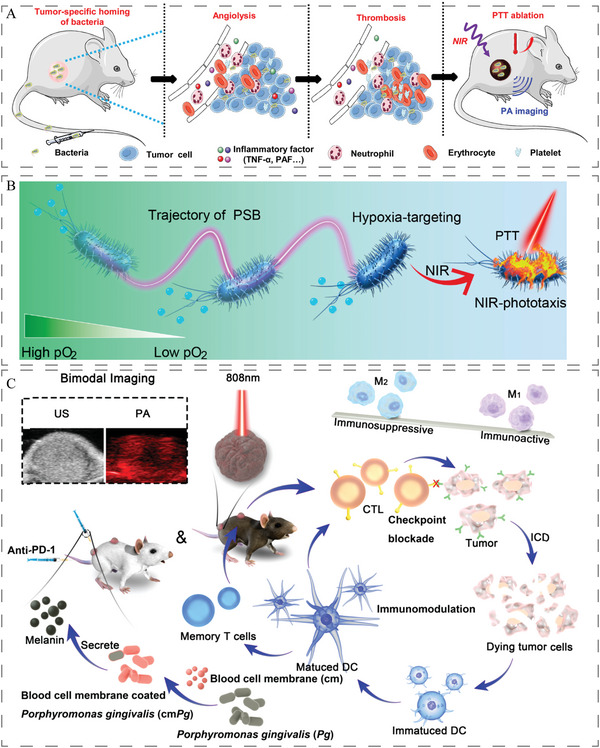
A) Schematic illustration of bacteria‐triggered tumor thrombosis and the subsequent photothermal tumor ablation. The enhanced NIR absorbance of the tumor is visualized by in vivo PA imaging. Reproduced with permission.^[^ ^170^ ^]^ Copyright 2020, The American Association for the Advancement of Science. B) Schematic showing that photosynthetic bacteria with self‐propelled and NIR‐phototaxic capacities can be utilized as photothermal agents for hypoxia‐targeted cancer elimination. Reproduced with permission.^[^ ^171^ ^]^ Copyright 2021, American Chemical Society. C) A scheme showing the fabrication procedure and working principle of cmPg that could secrete melanin and modulate tumor immune microenvironment. In B16F10‐bearing and CT26‐bearing mice, the internalized melanin produced by cmPg will be used for bimodal ultrasonic/photoacoustic (US/PA) imaging‐guided PTT of cancer. Moreover, upon 808 nm laser irradiation, disintegrated cmPg will induce the phenotype polarization of macrophages, causing M1‐like TAMs to outweigh M2‐like TAMs. When combined with anti‐PD‐1 immunotherapy, cytotoxic lymphocytes (CTLs) will recognize more tumor cells and exacerbate immunogenic cell death (ICD) comes from cancer cells. The antigens from dying tumor cells will be captured by immature DCs. Then immature DCs will transform into mature DCs. Mature DCs will effectively activate cellular immunity, which CTLs and memory T cells involve. On this basis, the growth of tumors could be inhibited. Reproduced with permission.^[^ ^148^ ^]^ Copyright 2021, Elsevier.

Bacteria can specifically colonize tumor tissues, activate innate immunity to release proinflammatory cytokines and destroy tumor blood vessels. The leaked blood can coagulate to form thrombi in the tumor tissue with strong NIR absorption ability. As shown in **Figure** **6**A, a few hours after intravenous injection of low doses of the △ppGpp (*S. typhimurium*), different types of solid tumors on mice would become obviously darkened.^[^ ^170^ ^]^ Live photosynthetic bacteria (PSBs) have been used as hypoxia‐targeting vehicles for hypoxic tumors due to their physiological properties as facultative aerobes. Due to its NIR and hypoxia chemotaxis, enabling targeted cancer PTT without late modification. Furthermore, as native bacteria, PSB can enhance the immune response, thereby inducing the infiltration of cytotoxic T lymphocytes (Figure 6B).^[^ ^171^ ^]^ *P. gingivalis* can stably secrete melanin as a photothermal agent during gingival and periodontal infections. Notably, *P. gingivalis* can promote the polarization of macrophages toward the M1 phenotype, which is required for tumor suppression. As shown in Figure 6C, the encapsulation of *P. gingivalis* through the erythrocyte membrane not only reduces the uptake of macrophages, but also stimulates the secretion of melanin by utilizing the heme in erythrocytes as a fertilizer for *P. gingivalis*.^[^ ^148^ ^]^

**Figure 7:**
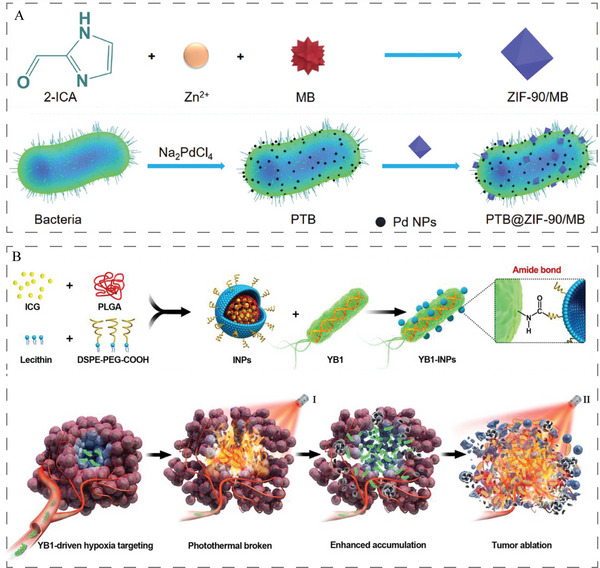
A) Schematic illustration of synthetic procedure of ZIF‐90/MB, and synthetic procedure of PTB and PTB@ZIF‐90/MB. Reproduced with permission.^[^ ^172^ ^]^ Copyright 2020, Wiley‐VCH. B) Preparation procedure of YB1‐INPs with hypoxia‐targeting and photothermal‐assisted bioaccumulation for tumor penetrative therapy. Reproduced with permission.^[^ ^92^ ^]^ Copyright 2019, Elsevier.

Subsequently, we focus on the application of bacteria as carriers of photothermal agents for PTT. As shown in **Figure** **7**A, the facultative anaerobic bacterium *Shewanella oneidensis* MR‐1 was engineered by surface biomineralizing palladium nanoparticles and conjugating methylene blue (MB)‐loaded ZIF‐90 (ZIF‐90/MB) to form a photothermal bacterium (PTB@ZIF‐90/MB) for tumor targeting PTT. PTB@ZIF‐90/MB induces mitochondrial dysfunction by inhibiting ATP generation and downregulating the expression of HSP70 and HSP90261, overcoming the great potential of tumor targeting and tumor thermotolerance challenges in PTT.^[^ ^172^ ^]^ Chen et al. used highly motile *S. typhimurium* strain YB1‐loaded NPs with the photosensitizer indocyanine greens (INPs) to target large bladder tumors (≥500 mm^3^). Among them, YB1 enhanced the penetration of INPs into the hypoxic part of tumors, and after NIR laser irradiation, INPs were able to achieve photothermal lysis of tumor cells (Figure 7B).^[^ ^92^ ^]^ Furthermore, *B. bifidum* (B.b) with hypoxia targeting ability was loaded with Ag_2_S quantum dots (QDs) via electrostatic interaction to prepare a hybrid bacteria with the abilities for tumor targeting and penetration, TAM polarization, and photothermal conversion (B.b@ QDs) to improve antitumor immunotherapy in vivo.^[^ ^173^ ^]^ In addition, the study also showed that perylenediimide derivative‐based supramolecular complexes (CPPDI) can be selectively reduced to radical anions (RAs) in hypoxic tumors depending on *E. coli* to achieve high‐precision PTT of tumors.^[^ ^143b^ ^]^

In summary, there are extensive applications of bacteria‐based PTT, which largely solves the problem of targeted delivery of photothermal agents to promote novel bacteria therapies for cancer treatment.

#### 3.1.6 Combined with Photodynamic Therapy

Photodynamic therapy (PDT) mainly utilizes ROS generated during photoactivation of photosensitizers (PSs) to induce apoptosis, necrosis, and tissue destruction, which is widely used in cancer treatment due to its less invasiveness, fewer side effects, and lower risk of drug resistance.^[^ ^150^ ^]^ Bacteria‐mediated PSs delivery addresses the limitations of PDT efficacy caused by the hypoxic TME where high oxygen consumption of PSs further exacerbates hypoxia at the tumor site.^[^ ^174^ ^]^

**Figure 8:**
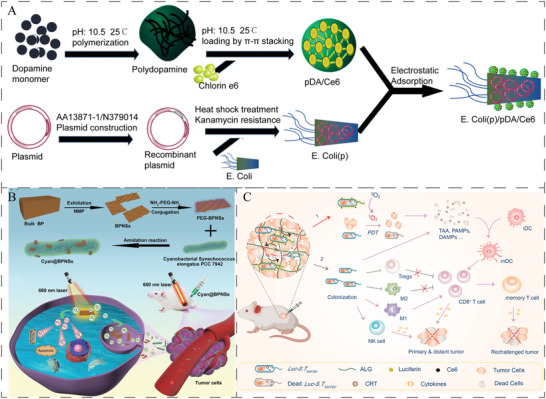
A) Schematic preparation of *E. Coli*(p)/pDA/Ce6. Reproduced with permission.^[^ ^175^ ^]^ Copyright 2021, Elsevier. B) Fabrication and photosynthesis‐promoted photodynamic therapy (PDT) schematics of Cyan@BPNSs. Reproduced with permission.^[^ ^176^ ^]^ Copyright 2021, Wiley‐VCH. C) A scheme illustrating the engineering of bioluminescent bacteria to boost PDT and antitumor immunity for synergistic cancer treatment. Reproduced with permission.^[^ ^11^ ^]^ Copyright 2022, Elsevier.

As shown in **Figure** **8**A, transgenic *E. coli* (*E. Coli*(p)) overexpressing human catalase catalyze H_2_O_2_ to O_2_ at the tumor site. The generated O_2_ was converted into cytotoxic ^1^O_2_ upon irradiation with NIR for photodynamic therapy. Polydopamine (pDA) was used to encapsulate photosensitizer Ce6 to form pDA/Ce6, which was used to coat *E. Coli*(p) to form *E. coli*(p)/pDA/Ce6. *E. coli*(p)/pDA/Ce6 were administered intravenously to selectively accumulate and replicate in hypoxic tumors while NIR light irradiation was introduced for photothermal and O_2_‐enhanced photodynamic therapy.^[^ ^175^ ^]^ Besides, photosynthetic cyanobacteria that continuously produced oxygen in situ through photosynthesis were modified by inorganic 2D black phosphorus nanosheets (BPNSs) to become a novel bioreactor called Cyan@BPNSs (Figure 8B). Under 660 nm laser irradiation, photosynthetic cyanobacteria continuously generated oxygen in situ through photosynthesis, and then photosensitization by BPNSs activated oxygen to singlet oxygen (^1^O_2_), which led to the accumulation of a large amount of ^1^O_2_ at the tumor site to kill tumor cells.^[^ ^176^ ^]^ However, the efficacy of aggregation‐induced emission (AIE)‐based PDT is limited by several factors including limited depth of laser penetration and intratumoral hypoxia. A novel *E. coli*‐based AIEgen (TBP‐2) hybrid system (AE) was thus developed to facilitate hypoxia‐tolerant PDT therapy for colon tumors in situ by an interventional approach^[^ ^177^ ^]^. Similarly, *E. coli* MG1655 was coated with a zeolitic imidazolate framework‐8 (ZIF‐8) layer coloaded with Ce6 and DOX through a one‐step in situ method to form *E. coli*@ZIF‐8/C&D. It exhibited high therapeutic efficacy both in vitro and in vivo in a combined chemo‐photodynamic manner.^[^ ^178^ ^]^ In addition, an engineered *E. coli*/BPQDs (EB) system was generated by electrostatic adsorption of catalase‐expressing *E. coli* onto black phosphorus quantum dots (BPQDs) also for photodynamic therapy.^[^ ^179^ ^]^ As shown in Figure 8C. Yang et al. engineered bioluminescent bacteria by transforming S.T.ΔppGpp with firefly‐luciferase‐expressing plasmid (Luc‐S.T.ΔppGpp) as an internal light source to evenly illuminate the whole tumor. Upon being fixed inside tumors with in situ formed hydrogel, the colonized Luc‐S.T.ΔppGpp together with luciferase substrate d‐luciferin could continuously generated light to excite photosensitizer chlorin e6 (Ce6), leading to effective suppression of different types of tumors including CT26 bearing tumor mouse, B16 bearing tumor mouse and VX2 bearing rabbit.^[^ ^11^ ^]^ Meanwhile, LucS.T.ΔppGpp‐enhanced PDT can also elicit potent antitumor immunity after treatment, thereby suppressing tumor metastasis and preventing tumor recurrence. Overall, coating living bacteria with PS‐containing nanoparticles not only provides an alternative strategy for optimizing bacteria‐mediated cancer therapy, but also offers promising opportunities for intracellular protein delivery.^[^ ^174^ ^]^

#### 3.1.7 Synergize Immunotherapy

**Figure 9:**
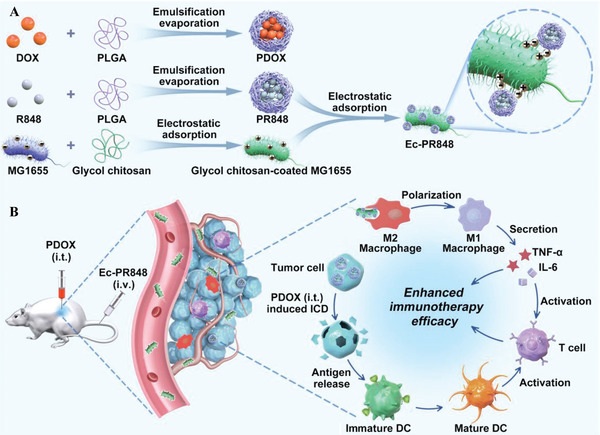
Schematic illustration of A) the preparation of PR848‐Loaded *E. coli* (Ec‐PR848) and B) ICD enhanced the efficacy of immunotherapy based on TAM polarization. A,B) Reproduced with permission.^[^ ^120c^ ^]^ Copyright 2021, American Chemical Society.

In addition to the aforementioned utilization of bacteria as biological carriers, the natural adjuvant components of bacteria can also stimulate immune maturation and induce inflammation in a controlled manner to increase tumor immune infiltration.^[^ ^180^ ^]^ As shown in **Figure** **9**, PLGA‐R848 (PR848) was attached to the surface of *E. coli* MG1655 by electrostatic adsorption to form nanoparticle/bacteria complexes (Ec‐PR848) for tumor‐targeting and tumor immunotherapy. The toll‐like receptor 7/8 (TLR7/8) agonist resiquimod (R848) and *E. coli* can together polarize M2 macrophages to M1 macrophages, while PDOX‐induced ICD can also impair the immunosuppression of the TME.^[^ ^120c^ ^]^

#### 3.1.8 For Modulating Microbiota

Microbiota plays a crucial role in cancer development and treatment. Studies show that although the microbiota is able to promote tumor growth, its presence also improves the efficacy of cancer treatment such as immunotherapy.^[^ ^181^ ^]^ For example, Antibiotics eliminate gut microbiota increased tumor vascular permeability, which allowed more LipoDox nanoparticles to accumulate in the tumor and augmented long‐term antitumor therapeutic efficacy.^[^ ^181^ ^]^ Phage or bioinorganic hybrid phage has been shown to regulate intestinal microbiota to remodel tumor‐immune microenvironment against colorectal cancer.^[^ ^51^, ^182^ ^]^ Oral administration of inulin, a polysaccharide dietary fiber found in chicory root and Jerusalem artichoke, improved the antitumor efficacy of anti‐PD‐1 (*α*‐PD‐1) immune‐checkpoint blocks therapy.^[^ ^183^ ^]^ In addition, biomaterials mediated oral microbiota regulation for enhanced immunotherapy of oral squamous cell carcinoma has also been reported.^[^ ^184^ ^]^ To date, oral bacteria mediated regulation of intestinal flora for the treatment of colitis is not uncommon.^[^ ^155^ ^]^ However, the research on oral bacteria mediated microbiota regulation for cancer treatment is rare, which may be a new idea for future cancer treatment.

#### 3.1.9 Others

**Figure 10:**
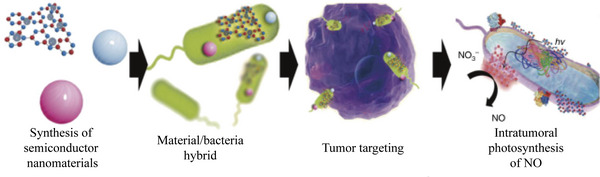
Schematic diagram of the preparation of PMT system. Reproduced with permission.^[^ ^185^ ^]^ Copyright 2018, Springer Nature.

Undoubtedly, the abundant proteins on the surface of bacteria endow it with multiple functions, which can develop various strategies for tumor therapy.^[^ ^166^ ^]^ As shown in **Figure** **10**. Zheng et al.^[^ ^185^ ^]^ propose a strategy of “charging” bacteria with a nano‐photocatalyst to strengthen their metabolic activities. For example, carbon nitride (C_3_N_4_) was combined with *E. coli* carrying nitric oxide (NO) generation enzymes for photo‐controlled bacterial metabolite therapy (PMT). Under light irradiation, photoelectrons produced by C_3_N_4_ could be transferred to *E. coli* to promote the enzymatic reduction of endogenous NO_3_— to cytotoxic NO with a 37‐fold increase. In CT‐26 tumor bearing mouse model, C_3_N_4_‐loaded bacteria were perfectly accumulated throughout the tumor, in which the PMT treatment resulted in ≈80% inhibition of tumor growth. For another example, Fan et al.^[^ ^143a^ ^]^ designed engineered *E. coli* MG1655 with overexpression of NDH‐2 enzyme (respiratory chain enzyme II) (Ec‐pE), which can colonize tumor mass and increase localized H_2_O_2_ generation. Thereafter, magnetic Fe_3_O_4_ nanoparticles were covalently linked to bacteria to act as a catalyst for a Fenton‐like reaction, which converted H_2_O_2_ to toxic hydroxyl radicals (•OH) to treat tumor. In this constructed bioreactor, the Fenton‐like reaction occurs with sustainably synthesized H_2_O_2_ produced by engineered bacteria, while severe tumor apoptosis is induced by toxic •OH products.

### 3.2 Gene Editing

#### 3.2.1 Genetic Modules That Have Been Used for Bacterial Genetic Engineering

As mentioned above, bacteria can be modified by chemical logistics for immune capture, drug, virus, and radionuclide delivery. These one‐time, static modifications of bacteria do not allow in situ modulation and can lead to uncontrolled growth, off‐target tissue toxicity or compromised cellular function, resulting in reduced efficacy. Moreover, unlike conventional drug carriers, the unique abilities of bacteria to continuously proliferate, translocate, and deliver therapeutic payloads in cancerous tissue necessitates robust and temporal control of bacterial pharmacokinetics in vivo.^[^ ^18b^ ^]^ Genetically engineered bacteria can strictly control the expression of related genes, which is crucial for managing the time and place of drug production.^[^ ^186^ ^]^ Precise trigger expression can increase drug concentration in tumor and minimize harmful side effects.^[^ ^111e^ ^]^ There are many interesting gene expression systems, such as quorum sensing,^[^ ^111^, ^186^ ^]^ l‐arabinose system,^[^ ^187^ ^]^ doxycycline induction,^[^ ^114b^ ^]^ etc.

The relative prevalence of individual species within a consortium can have major effects on the behavior of the community. Cooperation between strains, selective environments, engineering “self‐limiting” growth, and the spatial separation of strains can be achieved in a consortium through quorum sensing (QS) molecules.^[^ ^188^ ^]^ In order to control population levels and facilitate drug delivery using bacteria, Din et al. engineered a synchronized lysis circuit (SLC), consisted of a common promoter that drives expression of both its own activator (positive feedback) and a lysis gene (negative feedback). Specifically, the luxI promoter regulates production of autoinducer (AHL), which binds LuxR and enables it to transcriptionally activate the promoter. Negative feedback arises from cell death that is triggered by a bacteriophage lysis gene (*ϕ*X174 E), which is also under control of the luxI promoter. AHL can diffuse to neighboring cells and thus provides an intercellular synchronization mechanism.^[^ ^186^ ^]^

In addition, tumor‐specific expression of antitumor drugs can be achieved using engineering bacterium harboring the P_BAD_ promoter, which is induced by l‐arabinose.^[^ ^187b^ ^]^ For example, Yue et al. modified *E. coli* to express, under the control of a promoter induced by the monosaccharide arabinose, a specific tumor antigen fused with the protein cytolysin A on the surface of outer membrane vesicles (OMVs) released by the commensal bacteria.^[^ ^18a^ ^]^ Using an attenuated strain of ΔppGpp *S. typhimurium*, Jiang et al. engineered an inducible bacterial drug delivery system, in which coexpression of a therapeutic gene and a bioluminescence reporter gene occurred only in response to the administration of exogenous doxycycline in a dose‐dependent manner. This system enabled the repeated imaging of individual experimental animals in which both the therapeutic gene and reporter gene were coexpressed to achieve the desired biological effect.^[^ ^114b^ ^]^

#### 3.2.2 Attenuated Bacteria

Some bacteria may secrete some virulence factors, which can increase the risk of infecting the host during BMAT.^[^ ^104^ ^]^ To reduce this risk, gene editing technology is usually used to knock out the relevant genes expressing those virulence factors while retaining the therapeutic capabilities of the bacteria to become an attenuated bacterial strain.^[^ ^12^, ^95^, ^103^, ^111^, ^189^ ^]^

The intracellular bacterium *L. monocytogenes* has been recognized as a cancer vaccine platform because of its ability to induce potent innate and adaptive immunity.^[^ ^15^, ^102^ ^]^ A series of recombinant safer and potent live attenuated vaccine strains were then developed for clinical application.^[^ ^190^ ^]^ For example, by selectively deleting two virulence factors, ActA (△actA) and Internalin B (△inlB), the immunopotency of *Listeria* was kept while its toxicity was significantly reduced for in vivo application. In a mouse model, *Listeria* △actA/△inlB‐based vaccines were rapidly cleared after immunization but induced potent and durable effector and memory T‐cell responses with no measurable liver toxicity.^[^ ^102^ ^]^

Various attenuated strains of *S. typhimurium* have also been designed and engineered to be tumor‐targeting therapeutics or drug delivery vehicles, which showed both an excellent safety profile and therapeutic efficacy in mouse models.^[^ ^91a^ ^]^ VNP20009, an attenuated strain of *S. typhimurium* with purI and msbB genes deletions, was able to target and inhibit tumor growth in human‐NF2 schwannoma^[^ ^116^ ^]^ and PDAC mice.^[^ ^118^ ^]^ These findings led to the present phase I study of the intravenous infusion of VNP20009 to patients with metastatic cancer.^[^ ^95^ ^]^ ΔppGpp *Salmonellae*, a strain of *S. typhimurium* with a defect in guanosine 5′‐diphosphate‐3′‐diphosphate synthesis, inhibited the growth of CT26 or Hep3B2.1–7 tumors in mouse models.^[^ ^112^, ^114^ ^]^ A1‐R, a mutant of *S. typhimurium* with auxotrophic for leu‐arg and increased antitumor virulence selected by tumor passage, PC‐3 prostate human tumors and MARY‐X human breast tumors in nude mice.^[^ ^12^, ^111^ ^]^ Besides, a live strain of *Pseudomonas aeruginosa* was engineered to translocate a recombinant antigenic protein into dendritic cells (DCs), induced the activation of ovalbumin‐specific CD8^+^ T lymphocytes, and was resistant to a subsequent challenge with an ovalbumin‐expressing melanoma.^[^ ^191^ ^]^ Moreover, attenuated *E. coli* ^[^ ^19^ ^]^ and *C. novyi*‐NT^[^ ^97^ ^]^ were designed for the treatment of tumors. Therefore, live attenuated bacteria with deletion of specific virulence factors represent an attractive therapeutic platform for further development and investigation in clinical trials.

#### 3.2.3 Genetically Modified Bacteria with Specific Functions

In addition to knocking out virulence factors in bacteria through gene editing technology, genetic modification can also be used to increase their effectiveness. Injectable drug delivery systems that autonomously detect, properly infiltrate, and ultimately target tumor cells, are one of the future directions of precision medicine.^[^ ^159^ ^]^ Currently, there are four main gene editing strategies targeting bacteria for tumor therapy: 1) expression of antitumor cytokines,^[^ ^111b^ ^]^ 2) expression of immunomodulators,^[^ ^192^ ^]^ 3) expression of signaling proteins for imaging guide,^[^ ^120b^ ^]^ and 4) expression of prodrug‐converting enzymes.^[^ ^193^ ^]^

##### Expression of Antitumor Cytokines

Short half‐life in blood circulation, lack of tumor‐specific cellular uptake, and insufficient tumor exposure limit the application of antitumor cytokines for in vivo use,^[^ ^194^ ^]^ which makes nontargeted systemic administration not a feasible strategy on most occasions. Some bacteria can preferentially accumulate in TME, which can be manipulated to reduce the systemic cytotoxicity,^[^ ^195^ ^]^ such as *S. typhimurium*,^[^ ^111^, ^196^ ^]^ *L. monocytogenes*,^[^ ^197^ ^]^ *E. coli*,^[^ ^121^, ^169^ ^]^ etc. Thus, the development of engineered nonpathogenic bacteria that are capable of expressing antitumor proteins, is an ideal approach for selectively eradicating cancer cells.^[^ ^120^, ^187^ ^]^

**Figure 11:**
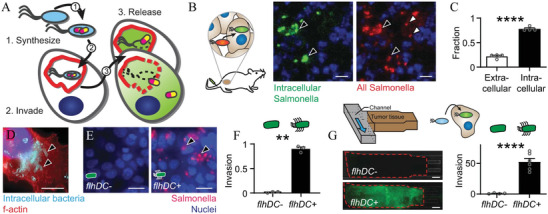
A) The design goals were to genetically engineer a bacterial vehicle that ① synthesizes a protein drug (yellow/purple), ② actively invades into cancer cells, and ③ releases drug, which escapes *S. typhimurium* vacuoles (SCVs, red). B,C) Ninety‐six hours after intratumoral injection of 2 × 10^6^ CFU of intracellular‐reporting *S. typhimurium* into subcutaneous 4T1 tumors in BALB/c mice, more bacteria (red) were intracellular (green; black arrows) than extracellular (white arrows; *P* < 0.0001). D) *S. typhimurium* (light blue, arrows) invade cancer cells (red). E) Knockout ΔflhD *S. typhimurium* were transformed with PBAD‐flhDC. Uninduced bacteria (flhDC−) minimally invaded cancer cells. Induction with 20 mm arabinose (flhDC+) promoted invasion (black arrows). F) Re‐expression of flhDC significantly increased invasion. G) In 3D tumor‐on‐a‐chip devices (top left), flhDC+ *S. typhimurium*, with a green invasion reporter (top right), invaded more cells than flhDC− controls. A‐G) Reproduced with permission.^[^ ^198^ ^]^ Copyright 2021, Springer Nature.

As shown in **Figure** **11**, Raman et al. designed nonpathogenic therapeutic *S. typhimurium* strain (ID *Salmonella*), which controls protein phosphatase 1 (NIPP1‐CD) and constitutive two‐chain active caspase‐3 (CT Casp‐3) protein synthesis, invasion into cells, and release. In mouse models of breast cancer and HCC, ID *Salmonella* were safe, decrease tumor growth, and reduce established breast metastases.^[^ ^198^ ^]^ *S. typhimurium* was also engineered to express the proapoptotic cytokine FasL, which would inhibit the growth of D2F2 tumors by an average of 59% and CT26 tumors by an average of 82%.^[^ ^111b^ ^]^ Engineered *S. typhimurium* could secrete murine TNF‐related apoptosis‐inducing ligand and induce caspase‐3‐mediated apoptosis of 4T1 breast carcinoma cells under the control of prokaryotic radiation‐inducible RecA promoter.^[^ ^195^ ^]^ Different antitumor cytokines have also been delivered to tumor tissues using engineered *Salmonella*, such as ClyA,^[^ ^112^, ^187^ ^]^ mitochondrial targeting domain of Noxa,^[^ ^113d^ ^]^ etc.

*Staphylococcus aureus* alpha‐hemolysin (SAH), coding a pore‐forming protein, was cloned into *E. coli* to kill cancer cells and suppress tumor growth.^[^ ^123^ ^]^ *E. coli* MG1655 transformed with plasmid pBV220 (containing a thermosensitive promoter with a therapeutic protein coding gene (TNF‐*α*)), as heat‐sensitive bacteria, could rapidly colonize tumor and induce the expression of TNF‐*α* under NIR.^[^ ^199^ ^]^ Similarly, the engineered *E. coli* MG1655 expressing ClyA was also designed for antitumor therapy.^[^ ^121^, ^169^ ^]^ Ho et al. reprogrammed commensal EcN to bind to the heparan sulfate proteoglycan on the cancer cell surface and to secrete myrosinase for the conversion of dietary glucosinolate to sulforaphane. The produced sulforaphane inhibits growth and promotes apoptosis in cancer cells, resulting in colorectal tumor clearance.^[^ ^121b^ ^]^

Engineered *L. monocytogenes* can also express antitumor cytokines for cancer therapy. For instance, a genetic modification of the replication‐deficient *L. monocytogenes* strain DdalDdat (Lmdd) secreting human CD24 protein has been developed, which effectively increased the number of CD8^+^ T cells and T helper 2 cells and promoted IFN‐*γ* secretion, significantly reducing Hepa1–6‐CD24 tumor size and increasing the mice survival.^[^ ^106^ ^]^ In addition, *L. monocytogenes* was able to deliver tetanus toxoid protein to pancreatic tumors to induce cancer cell death in mice.^[^ ^107^ ^]^

##### Expression of Immunomodulators

Although immunomodulators have revolutionized cancer treatment, they can also cause many immune‐related side effects such as fatigue, rash, endocrine disorders, and liver toxicity,^[^ ^200^ ^]^ which may lead to eventual discontinuation of the treatment.^[^ ^201^ ^]^ Fortunately, the advances in genetic technology and synthetic microbiology have enabled to engineer some smart microbial delivery systems to improve the therapeutic application of immunomodulators.^[^ ^201^ ^]^

**Figure 12:**
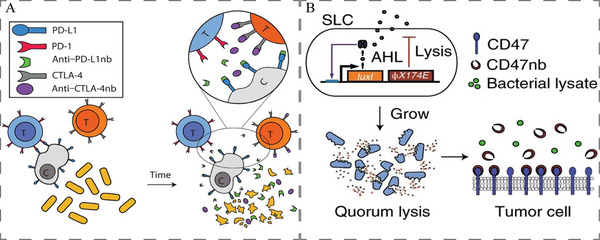
A) Schematic showing the mechanism by which engineered bacteria controllably release constitutively produced PD‐L1 and CTLA‐4 blocking nanobodies intratumorally. Reproduced with permission.^[^ ^201^ ^]^ Copyright 2020, The American Association for the Advancement of Science. B) *E. coli* with SLC reach a quorum and induce the phage‐lysis protein *ϕ*X174E, leading to bacterial lysis and release anti‐CD47 blocking nanobody. Reproduced with permission.^[^ ^125^ ^]^ Copyright 2019, Springer Nature.

As shown in **Figure** **12**A, EcN were engineered to control the production and release of nanobodies targeting PD‐L1 and cytotoxic T lymphocyte‐associated protein‐4 (CTLA‐4) using a stabilized lysing release mechanism.^[^ ^201^ ^]^ Similarly, a nonpathogenic *E. coli* strain that can specifically lyse and release a CD47 nanoantagonist (CD47nb) in TME was designed by Chowdhury et al., which increased the activation of tumor‐infiltrating T cells, stimulated rapid tumor regression, prevented metastasis, and led to long‐term survival in A20 B cell lymphoma mouse model (Figure 12B).^[^ ^125^ ^]^ Yoon et al. fused IFN*γ* to the N‐terminal region of SipB by overexpressing IFN*γ* in attenuated *S. typhimurium* (*S. typhimurium* (IFN‐*γ*)), which effectively suppressing B16F10 melanoma tumor growth and prolonging the mice survival.^[^ ^202^ ^]^ Besides, FlaB,^[^ ^115^ ^]^ CCL21,^[^ ^111c^ ^]^ IL‐2,^[^ ^203^ ^]^ IL‐18,^[^ ^196^ ^]^ etc. have also been used as immunomodulators delivered by bacteria for cancer therapy.

##### Expression of Signaling Proteins

In recent years, theranostic agents that integrate imaging and therapeutic functions have been popular in the research for antitumor therapy.^[^ ^204^ ^]^ Optical imaging techniques based on bacteria‐mediated bioluminescence and fluorescence can not only detect successful bacteria localization in tumors to predict their proliferation, but also improve the diagnosis and treatment of cancers.^[^ ^20^ ^]^ For example, *E. coli* MG1655 engineered to express tyrosinase, which uses l‐tyrosine as the substrate to produce the strong optoacoustic probe melanin^[^ ^205^ ^]^ or carried ^18^F‐FDS PET to visualize CT‐26 tumor in mouse model.^[^ ^20b^ ^]^ *S. typhimurium* have also been engineered to express bacterial luciferase gene lux,^[^ ^112a^ ^]^ RLuc8,^[^ ^187b^ ^]^ or GFP^[^ ^110^, ^111^, ^112^ ^]^ for visualization of tumor distribution.^[^ ^118^ ^]^ These imaging technologies have enabled real‐time monitoring of bacterial migration into both primary and metastatic tumors in different mouse tumor models.^[^ ^111^, ^206^ ^]^

##### Expression of Prodrug‐Converting Enzymes

The bacterial‐based prodrug‐converting enzymes delivery system utilizes the tumor‐specific infiltration of bacteria to selectively introduce gene encoding prodrug‐activating enzymes into tumor cells, realizing the local activation of “prodrug” within the tumor.^[^ ^109^, ^207^ ^]^ Royo et al. integrated a regulatory control circuit activated by acetyl salicylic acid (ASA) in attenuated *Salmonella enterica aroA* (SL7207‐4S2 strain) (*S. enterica*) that carries an expression module encoding the 5‐fluorocytosine (5‐FC)‐converting enzyme cytosine deaminase in the bacterial chromosome or in a plasmid. Local expression of cytosine deaminase was induced by ASA after administration of *S. enterica* to fibrosarcoma mice.^[^ ^208^ ^]^ Prodrug‐activating enzyme carboxypeptidase G2 (CPG2) was also delivered to MDA‐MB‐361 (human breast carcinoma) tumors, WiDr (human colon carcinoma) tumors, or B16‐F10 tumors by VNP20009.^[^ ^109b^ ^]^ Interestingly, studies have shown that some probiotics can achieve high levels of activation of various prodrugs, especially at the site of action, without the need for genetic modification.^[^ ^209^ ^]^

##### Others

Bacterial‐based antiangiogenic drug delivery systems are expected to target tumor tissues to inhibit neovascularization, vascular remodeling, tumor growth, and metastasis.^[^ ^210^ ^]^ In addition, metabolites (such as short‐chain fatty acids (SCFAs),^[^ ^211^ ^]^ bile acids,^[^ ^212^ ^]^ butyrate,^[^ ^213^ ^]^ and lysine^[^ ^213^ ^]^) derived from bacterial energy metabolism in TME, altered host metabolites, and other bacterial products can constantly interact with host metabolism. These metabolites can also modulate microbial abundance and function medicated by immune homeostasis. Therefore, engineered microbial therapy is capable of modulating the metabolism of the TME, thereby enhancing the efficacy of immunotherapy.^[^ ^122^ ^]^

### 3.3 Antitumor Strategies Based on Bacterial Components and Products

Different species of bacteria, alone or in combination with other therapies, have shown incredible potential to infiltrate and colonize solid tumors leading their suppression. Besides, different bacterial products such as OMVs, peptides, toxins, enzymes, and spores can be programmed and designed to sense and respond to environmental signals to deliver therapeutic effectors.^[^ ^21c^ ^]^ The following section focuses on bacterial‐derived components and products for selective tumor‐targeted delivery of compounds, genetic materials, and/or other therapeutics.

#### 3.3.1 OMVs

**Figure 13:**
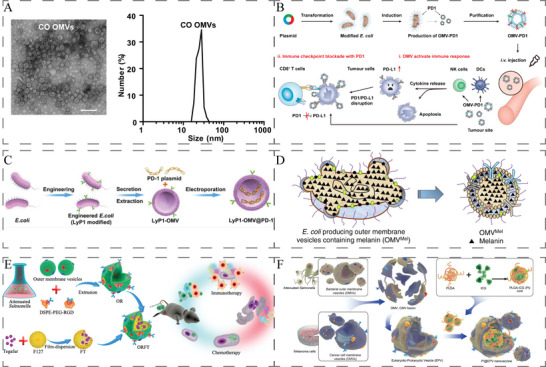
A) TEM image and DLS analysis of ClyA‐OVA OMVs (CO OMVs). Scale bar: 100 nm. Reproduced with permission.^[^ ^214^ ^]^ Copyright 2021, Springer Nature. B) OMV‐PD1 was obtained by engineering *E. coli* to stably express the mouse PD1 ectodomain fused with the surface protein ClyA, and then purifying the vesicles from the parent bacteria by ultracentrifugation. OMV‐PD1 accumulation at the tumor site increases the infiltration of immune cells and activates immune response. At the same time, the PD1 ectodomain on the OMV‐PD1 urface blocks the PD1/PD‐L1 interaction and protects CD8^+^ T cells, which can then attack tumor cells. Reproduced with permission.^[^ ^216^ ^]^ Copyright 2020, American Chemical Society. C) The preparation process of LOMV@PD‐1 nanoparticle. Attenuated *E. coli* was genetically engineered to express tumor‐targeted peptide LyP1. Then, the OMVs were extracted and loaded with PD‐1 plasmid by electroporation to fabricate LOMV@PD‐1. Reproduced with permission.^[^ ^217^ ^]^ Copyright 2022, Wiley‐VCH. D) A schematic representation of OMV^Mel^ purified after vesiculation from the parental bacteria. Reproduced with permission.^[^ ^219a^ ^]^ Copyright 2019, Springer Nature. E) Schematic illustration of the bioengineering process of functionalized OMV‐coated polymeric micelles. Reproduced with permission.^[^ ^220^ ^]^ Copyright 2020, American Chemical Society. F) Schematic illustration of fabrication of eukaryotic–prokaryotic vesicles coated PI@EPV nanovaccine. Reproduced with permission.^[^ ^223^ ^]^ Copyright 2020, Wiley‐VCH.

Bacterial OMVs are natural nanoparticles with a diameter of 30–250 nm (**Figure** **13**A), secreted by Gram‐negative bacteria.^[^ ^18^, ^214^ ^]^ Compared to the weakened bacteria, OMVs are safer because the preparation is acellular.^[^ ^214^ ^]^ In the past several years, OMVs have been widely investigated as biotherapeutics and/or biomimetic carriers.^[^ ^215^ ^]^

First, OMVs contain many natural adjuvant components inherited from the parent bacterium but in a nonreplicative form, which can stimulate immune maturation and initiate inflammation in a controllable manner.^[^ ^180^ ^]^ As shown in Figure 13B, the engineered OMV‐PD1 specifically expressing PD1 was isolated and purified from *E. coli* that specifically expressing PD1. OMV‐PD1 could bind to PD‐L1 on the surface of tumor cells, promote the internalization and reduction of PD‐L1, and protect T cells from immunosuppression. OMV‐PD1 drove the accumulation of effector T cells in tumor and inhibited tumor growth through the combination of immune activation and checkpoint suppression.^[^ ^216^ ^]^

Second, bacterial OMVs have been extensively studied as delivery vehicles or vaccines.^[^ ^144^, ^214^ ^]^ For instance, Yue et al. designed engineered *E. coli* by transfecting plasmid expressing ClyA (a surface protein on OMVs) fused with a tumor antigen and Fc fragment of mouse IgG (ClyA–Ag–mFc). Controllable in situ production of tumor antigen‐loaded OMVs (OMV–Ag–mFc) in the gut was achieved by oral administration of modified bacteria and the expression inducer Ara. Tumor antigen‐specific immune activation resulted in significant inhibition of tumor growth and resistance to tumor recurrence in multiple mouse cancer models.^[^ ^18a^ ^]^ Similarly, Pan et al. developed bacterial OMVs modified with LyP1 polypeptide containing PD‐1 plasmid (LOMV) to induce self‐blocking of PD‐L1 in tumor cells (Figure 13C).^[^ ^217^ ^]^

Similar to bacteria, bacterial OMVs can be used to PTT by stimulating the secretion of tumor‐associated cytokines and cause the extravasation of red blood cells within the tumor, which led to significantly darker tumors.^[^ ^215^, ^218^ ^]^ Encapsulating the biopolymer melanin in *E. coli* OMVs expressing tyrosinase transgenes (OMV^Mel^) could not improve the photothermal properties of melanin, but also enhance immunotherapy (as shown in Figure 13D).^[^ ^219^ ^]^ Zhai et al. constructed biomimetic nanocarriers (PLOVs) by fusing attenuated *Salmonella* OMVs with photothermally sensitive liposomes (PTSLs). PLOVs promoted immune maturation and improved the tumor immune infiltration in vivo due to the thermal effect on OMVs self‐adjuvant and PTSLs under NIR irradiation.^[^ ^180^ ^]^

Furthermore, bacterial OMVs can also be combined with chemotherapy. Chen et al. designed a pH‐sensitive drug delivery system (siRNA@M‐/PTX‐CA‐OMVs) that used OMVs to load PTX, which could regulate the development of DNA damage response 1(Redd1)‐siRNA and target different components in TME. Studies have shown that siRNA@M‐/PTX‐CA‐OMVs released PTX for the first time in response to tumor pH.^[^ ^21b^ ^]^ As shown in Figure 13E, attenuated *Salmonella* OMVs were functionalized with contamination‐free polyethylene glycol and tumor‐targeting ligand Arg–Gly–Asp peptides to improve their retention in the circulation and tumor‐targeting ability. It was subsequently encapsulated on tegafur‐loaded polymer micelles (FT) to enhance the synergistic therapeutic effect of OMVs‐induced immunotherapy and chemotherapy.^[^ ^220^ ^]^

Co‐immunization with bacterial OMVs and tumor‐derived cell membranes (mTs) could elicit tumor regression.^[^ ^221^ ^]^ As well, hybrid bacterial OMVs and mTs to form novel functional vesicles (mTOMVs) can enhance innate immune responses for personalized immunotherapy.^[^ ^222^ ^]^ As shown in Figure 13F, Chen et al. designed and constructed a eukaryotic‐prokaryotic vesicle (EPV) nanoplatform by fusing melanoma cell membrane vesicles and attenuated *Salmonella* OMVs to enhance the function and scalability of OMVs. In a melanoma model, the poly(lactic‐*co*‐glycolic acid)–ICG moiety (PI)‐implanted EPV (PI@EPV) in combination with localized PTT with durable immune inhibition showed synergistic antitumor effect as a therapeutic vaccine.^[^ ^223^ ^]^

#### 3.3.2 Bacterial Cytoplasmic Membranes

**Figure 14:**
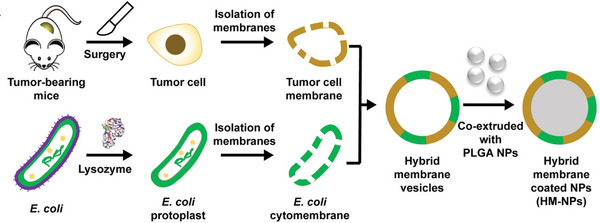
Preparation of HM‐NPs. Tumors were surgically removed from tumor bearing mice to obtain TMs. Amplified *E. coli* DH5*α* was treated with lysozyme to remove the cell wall, and an extraction buffer was used to prepare EMs. HM vesicles were generated by mixing EMs and TMs and extruding through an extruder. PLGA NP polymeric cores were then added, and repeated physical extrusion through the extruder was performed to generate HM‐NPs. Reproduced with permission.^[^ ^224^ ^]^ Copyright 2021, The American Association for the Advancement of Science.

Bacterial cytoplasmic membranes are double‐layer membrane nanovesicles derived from bacteria, which constitute the whole membrane and contain endogenous cellular targeting ligands.^[^ ^85^ ^]^ As shown in **Figure** **14**, Lin and co‐workers developed an antigen and adjuvant codelivery nanoparticle vaccine (HM‐NPs) based on *E. coli* cytoplasmic membranes (EMs) and tumor cell membranes (TMs) from resected autologous tumor tissue. HM‐NPs showed maximizing antitumor effects while avoiding side effects in CT26 colon, 4T1 breast cancer, B16F10 melanoma, and EMT6 breast tumor mouse models.^[^ ^224^ ^]^

#### 3.3.3 Bacterial Toxin

Most of the anticancer effects of bacteria are due to the toxins, which can target and destroy host cells and tissues.^[^ ^9b^ ^]^ For example, Listeriolysin O (*Lm*‐LLO) from *L. monocytogenes* can promote T cell viability and enhance tumor immunotherapy by inhibiting MDSC and Treg in TME,^[^ ^104^ ^]^ which were mainly associated with decreased expression of arginase I in MDSCs and IL‐10 in Tregs. *Pseudomonas aeruginosa* produces *Pseudomonas* exotoxin A (PEA) and exotoxin T (ExoT). PEA can be delivered into the cytosol by receptor‐mediated endocytosis, which induced a secondary immune response by activating T cells in addition to its intrinsic cytolytic activity.^[^ ^225^ ^]^ ExoT can cause tumor cell growth arrest by inhibiting the G1/S checkpoint protein.^[^ ^226^ ^]^ IL4‐Pseudomonas exotoxin (NBI‐3001) appeared to have an acceptable safety and toxicity profile when administered intratumorally in patients with recurrent malignant glioma.^[^ ^227^ ^]^ Moreover, *C. perfringens* enterotoxin, a pore‐forming (oncolytic) toxin, can induce selective cytotoxicity by binding to claudin‐3 and ‐4 (Cldn3/4).^[^ ^228^ ^]^

#### 3.3.4 Spore

As the dormant life form of bacteria, spores are highly resistant to the living environment and can be used for tumor therapy.^[^ ^229^ ^]^ In 1978, it was found that *C. perfringens* type A spores and their culture supernatant could prevent the growth of Ehrlich solid tumor.^[^ ^16^ ^]^ Subsequently, it was shown that *C. novyi*‐NT spores can germinate within avascular regions and hypoxic regions of tumors.^[^ ^128^ ^]^ *C. novyi*‐NT spores generate potent antitumor effect in experimental animal models,^[^ ^230^ ^]^ even in human patient who had an advanced leiomyosarcoma.^[^ ^97^ ^]^ Intratumoral injection of *C. novyi*‐NT spores result in increased phagocytosis and NK cell‐like function after treatment. Intravenous injection of *C. novyi*‐NT spores resulted in increased LPS‐induced TNF‐*α* production, LTA‐induced IL‐10 production and NK cell‐like function post‐treatment.^[^ ^231^ ^]^ In addition, spore coat could be converted into multifunctional coat nanoparticles (CN) by mechanical extrusion in vitro, which could repair epithelial barrier, inhibit IL‐1*β* and TNF‐*α*, and block IL‐6‐STAT3 signaling to prevent the development of CA‐CRC.^[^ ^229a^ ^]^

Similar to bacteria, spores can also be applied to combinate with nanomaterials. Prebiotic‐encapsulated probiotic spores (spores‐dex) were prepared by coating prebiotic glucan outside *Clostridium butyricum* spores through *β*‐cyclodextrin (*β*‐CD) and adamantane (AD)‐mediated host–guest interaction.^[^ ^232^ ^]^ After oral administration, spores‐dex specifically enriched in colon cancer, modulated gut microbiota, and increased the abundance of anticancer SCFA‐producing bacteria.

#### 3.3.5 Minicell

**Figure 15:**
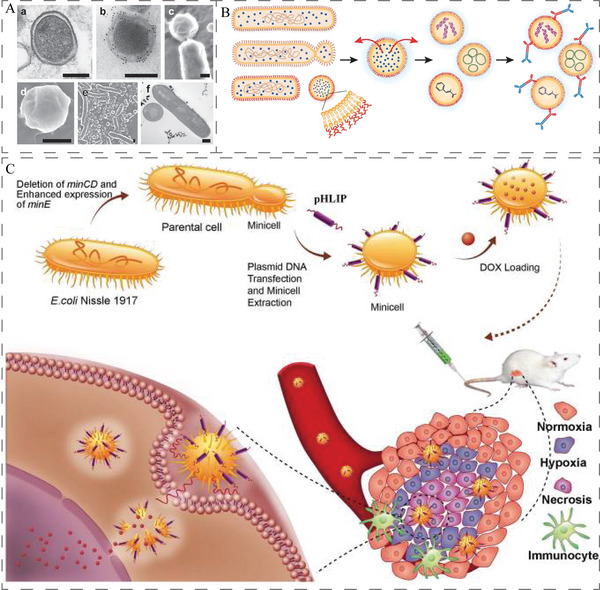
A) Transmission (Aa, Ab, and Af) and scanning electron microscopy (Ac–Ae) of minicells. Aa. minicells derived from *S. Typhimurium* showing inner and outer membrane structure; Ab. minicells derived from *S. Typhimurium* with immunogold labeling of surface O‐antigen; Ac. minicell budding off parental *E. coli*; Ad, S. flexneri minicell; Ae. mix of bacteria and minicells; Af. *L. monocytogenes* minicell and parent cell. Scale bar: 200 nm. Reproduced with permission.^[^ ^158b^ ^]^ Copyright 2007, Elsevier. B) Minicells are achromosomal particles produced during the division of mutant *S. typhimurium*. Their lipid bilayers are decorated with polysaccharides (red). Once emptied of their contents, minicells can be loaded with siRNAs (purple) or chemotherapeutics (black) by incubation in the appropriate solution. Plasmids encoding shRNA (green) can be introduced by transforming the mutant bacteria that generate the minicells. Loaded minicells are then functionalized via bispecific antibody conjugates, with one arm specific for sugars on the minicell surface (red) and the other specific for a targeting antigen or receptor (blue). Reproduced with permission.^[^ ^236^ ^]^ Copyright 2009, Springer Nature. C) Schematic illustration depicting the construction of the minicells^pHLIP^ for targeted delivery of chemotherapeutic drugs into the hypoxic regions of solid tumors to kill cancer cells. Reproduced with permission.^[^ ^234^ ^]^ Copyright 2018, Ivyspring International Publisher.

Minicells are 400 ± 20 nm anucleate nanoparticles (as shown in **Figure** **15**A) generated by inactivation of the genes that control normal bacterial cell division at the equatorial septation site.^[^ ^233^ ^]^ Exception of chromosomal DNA, these minicells contain the same cytoplasmic components as their parental bacteria^[^ ^234^ ^]^ and have been shown to be ideal tumor‐targeted drug delivery platform.^[^ ^235^ ^]^

As shown in Figure 15B, mutant *S. typhimurium* bacteria‐derived minicells were used to deliver siRNA, plasmid‐encoded shRNA targeting or the cytotoxic drug.^[^ ^236^ ^]^ Bispecific antibody‐targeted minicells, packaged with the chemotherapeutic paclitaxel, has entered phase I clinical trial without increasing proinflammatory factors, showing robust antitumor activity.^[^ ^233^ ^]^ A pH (low) insertion peptide (pHLIP) was displayed on the membrane surface of EcN minicells (minicells^pHLIP^) through protein display technology to endow the cells with the ability to target acidic TME (Figure 15C). DOX‐loaded minicells^pHLIP^ could successfully infiltrate the necrotic and hypoxic regions of orthotopic breast cancers and release drug in acidic media.^[^ ^234^ ^]^

#### 3.3.6 Others

Many enzymes secreted by bacteria are another products with tumor‐suppressing abilities, such as l‐asparaginase^[^ ^237^ ^]^ from *E. coil*. In addition, utilization of heat‐killed bacteria and bacterial proteins secreted in bacterial culture media can significantly reduce possible toxicity and infection risk.^[^ ^238^ ^]^

## 4 Conclusions and Future Directions

Nowadays, the role of bacteria in the occurrence, development, and treatment of tumors has already attracted much attention. Due to motility, chemotaxis, selective targeting, cytotoxic activities, immune regulatory, capacity as targeted delivery vehicles, and feasibility of design and modification, bacteria have been widely used in tumor therapy as therapeutic agents. In order to fight against virulence factors and harmful metabolites of bacteria, genetic engineering, and biosynthesis technology also emerged. This article mainly reviews the role of bacteria in the occurrence, development of cancer, and the latest progress of gene engineering and bacterial based bioactive materials in targeted delivery, antitumor, and antitumor immune precise treatment.

Although BMAT has made considerable progress in the field of cancer treatment, there are still many serious challenges for future clinical applications. 1) Universality. The targeting of bacteria to tumors is mainly due to hypoxia tropism, but can bacteria actively target tumor tissue when the tumor volume is small and the complete hypoxic microenvironment is not formed in the early stage of tumor? Preclinical studies have mainly focused on subcutaneous tumors. Are subcutaneous tumors and orthotopic tumors equally sensitive to BMAT? In addition, there are different mico‐ecology between animals and humans, and the results from various preclinical experiments can be translated into clinical application? 2) Stability. Unlike conventional drug carriers, the unique abilities of bacteria to continuously proliferate, translocate, and deliver therapeutic payloads in cancerous tissue necessitate robust and temporal control of bacterial pharmacokinetics in vivo. How to control gene mutation, antigen loss, and denaturation that may be caused in the process of genetic modification? How to avoid the loss of cargo due to the growth and reproduction of bacteria? 3) Biosecurity. How to effectively control the immune response caused by bacteria? Such as bacteremia, cytokine storm, etc. 4) Therapeutic efficacy. The transportation, enrichment, and function of engineering bacteria are limited by the temperature, pH value, nutrient source, etc. Therefore, the key is to ensure the maximum efficacy under the safe dose.

In conclusion, according to the idea of “harmony between human and nature” in traditional Chinese culture, maintaining the dynamic balance of the human ecosystem is more conducive to improve the immune system and overcome various unfavorable factors for cancer treatment. Therefore, both experimental and clinical in‐depth investigation in the development of novel BMAT originated from not only host commensal microbiota but also other microorganisms should be warranted in the future, hoping that human can benefit from the gifts from the nature to conquer cancer.

## Conflict of Interest

The authors declare no conflict of interest.
